# TG/HDL‐c ratio as a predictor of progressive infarction in patients with anterior circulation single subcortical infarction

**DOI:** 10.1002/brb3.3453

**Published:** 2024-02-26

**Authors:** Jing Lin, Liangbin Dong, Qin Huang, Hui Xiao, Shumeng Li, Jincai Tang, Xiaocheng Mao, Pengcheng Huang, Xiaobing Li, Daojun Hong

**Affiliations:** ^1^ Department of Neurology The First Affiliated Hospital of Nanchang University Nanchang Jiangxi China; ^2^ Department of Neurology Gaoxin Branch of The First Affiliated Hospital of Nanchang University Nanchang Jiangxi China

**Keywords:** anterior circulation single subcortical infarction, early neurological deterioration, insulin resistance, pontine single infarction, progressive infarction, triglyceride to high‐density lipoprotein cholesterol ratio

## Abstract

**Background:**

The contributors predicting progressive infarction (PI) in patients with anterior circulation single subcortical infarction (ACSSI) and pontine single infarction (PSI) may be unidentical. The role of triglyceride to high‐density lipoprotein cholesterol (TG/HDL‐c) ratio on PI is unclear. The purpose of our study is to evaluate the correlation between TG/HDL‐c ratio and PI in patients with ACSSI or PSI.

**Methods:**

Between January 2020 and October 2022, we retrospectively enrolled 738 patients including 638 ACSSI patients and 100 PSI patients to analyze. Demographic characteristics, clinical information, and laboratory data were collected within 24 h of admission.

**Results:**

PI occurred in 143 (19.4%) patients. In univariate analysis, patients with PI had higher initial National Institutes of Health Stroke Scale (NIHSS) scores, higher discharge NIHSS scores, higher levels of fasting glucose, total cholesterol, TG, low‐density lipoprotein cholesterol, and TG/HDL‐c ratio, but lower levels of creatinine compared to patients with non‐PI (*p* < .05). Furthermore, the results of the subgroup analyses revealed the independent association between TG/HDL‐c ratio and PI in ACSSI patients (OR 1.079, 95% CI 1.009–1.153, *p* = .026) rather than in PSI patients. Additionally, a receiver operating characteristic curve indicated that the optimal predictive cutoff value of the TG/HDL‐c ratio was 3.985, and a TG/HDL‐c ratio ≥3.985 was more likely to experience PI in ACSSI patients.

**Conclusion:**

In conclusion, the TG/HDL‐c ratio was independently associated with PI in patients with ACSSI.

## Graphical abstract

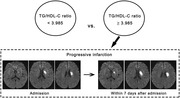

## 1 INTRODUCTION

Acute ischemic stroke, as the most common subtype of stroke, has become a leading cause of death and disability worldwide (Tsao et al., 2022). Single subcortical infarction (SSI), accounting for one‐third of all ischemic strokes, is related to a high possibility of early neurological deterioration (END), especially progressive motor deficits (Terasawa et al., 2008). Unlike watershed cerebral infarction where neurological worsening is mainly due to hemodynamic abnormalities, END in SSI may be caused by progressive occlusion of the proximal segment of a perforating artery or distal‐to‐proximal clot propagation with subsequent occlusion of small branches (Steinke & Ley, 2002; Yamamoto et al., 2011). More importantly, a variety of mechanisms have been proposed to account for END including progressive infarction (PI), increased intracranial pressure, recurrent cerebral ischemia, secondary parenchymal bleeding, and others (Siegler & Martin‐Schild, 2011). PI, representing an extension of the existing infarction, is the most common type of END, accounting for 33.6% (Siegler & Martin‐Schild, 2011). Although recent studies have demonstrated various predictive factors of END, different mechanisms may be responsible for END in different etiologic subtypes and lesion locations (Gong et al., 2020; Yamamoto et al., 1998). Thus, the contributors predicting END in anterior circulation SSI patients and posterior circulation SSI patients are not consistent (Nam et al., 2021). Additionally, END is a symptomatic diagnosis that covers multiple mechanisms, and the predictive factors for different mechanisms may vary. Specifically focusing on PI rather than END may improve the specificity and accuracy of the predictive factors.

There has been increasing evidence demonstrating that insulin resistance (IR) plays a crucial role in cardiovascular diseases and cerebrovascular diseases (Ormazabal et al., 2018; Yang et al., 2023). The triglyceride to high‐density lipoprotein cholesterol (TG/HDL‐c) ratio has been proposed as a credible surrogate indicator of IR because it shows strong correlations with the gold standard hyperinsulinemic‐glycemic clamp (Giannini et al., 2011). More importantly, the TG/HDL‐c ratio is more convenient and cost‐effective compared to some other IR indicators (Chen et al., 2022). In addition, the TG/HDL‐c ratio is identified as a superior predictor of metabolic syndrome in some ethnic populations (Behiry et al., 2019; Liang et al., 2015). Nam et al. (2019) demonstrated that TG/HDL‐c ratio was positively associated with the prevalence of silent brain infarction in a neurologically healthy population. Additionally, Park et al. (2014) revealed that an elevated TG/HDL‐c ratio was associated with recurrent stroke. However, the relationship between TG/HDL‐c ratio and END or PI is unclear.

Based on the following reason, anterior circulation SSI (ACSSI) and pontine single infarction (PSI) are the most common types of SSI, which usually develop PI. The purpose of this study is to evaluate the relationship between TG/HDL‐c ratio and PI in patients with ACSSI or PSI.

## 2 MATERIALS AND METHODS

### 2.1 Study populations and definitions

Between January 2020 and October 2022, we retrospectively assessed patients who were admitted to the Stroke Unit of First Affiliated Hospital of Nanchang University. All procedures were approved by the Ethics Committee of First Affiliated Hospital of Nanchang University. Patients were recruited if they met the following criteria: (1) admitted to our hospital and completed the first diffusion‐weighted imaging (DWI) within 48 h after symptom onset; (2) diagnosed as ACSSI or PSI on DWI consistent with the clinical deficits; (3) PI should be confirmed by DWI or computerized tomography (CT). Patients were excluded if they met the following criteria: (1) received intravenous thrombolysis or endovascular therapy; (2) suspected of cardiogenic embolism, arterial to arterial embolism, other determined etiology (moyamoya disease, arterial dissection, vasculitis, and so on); (3) neurological deficits worsening occurring before the first DWI; (4) lacked complete imaging, laboratory, or follow‐up data; and (5) the single infarct in medulla oblongata, midbrain, or thalamus. ACSSI is identified as the single subcortical infarct supplied from the lenticulostriate artery, Heubner's artery, or anterior choroidal artery (AchA) (Petrone et al., 2016). PSI is defined as the pontine single infarct supplied from the paramedian branches, short circumferential branches, or long circumferential branches (Petrone et al., 2016). As a main outcome, PI was defined as an increase of ≥1 point in motor power or ≥2 points on the total National Institutes of Health Stroke Scale (NIHSS) within 7 days after admission, and extension of the original infarction was further confirmed by DWI or CT (Li et al., 2023; Lin et al., 2022).

### 2.2 Data collection

We recorded the demographic characteristics and clinical information including age, sex, history of hypertension, diabetes, and stroke, initial NIHSS, discharge NIHSS, and infarction location. Laboratory data, including white blood cell (WBC), red blood cell (RBC), hemoglobin (HGB), platelet (PLB), blood urea nitrogen (BUN), creatinine, uric acid, fasting glucose, total cholesterol (TC), TG, HDL‐c, low‐density lipoprotein cholesterol (LDL‐c), fibrinogen, and D‐dimer, were routinely examined within 24 h of admission. The neutrophil‐to‐lymphocyte ratio (NLR), lymphocyte‐to‐monocyte ratio (LMR), and platelet‐to‐lymphocyte ratio (PLR) were calculated. TG/HDL‐c ratio was calculated using the following formula: TG/HDL‐c ratio = TG (mg/dL)/HDL‐c (mg/dL) (Park et al., 2014). All patients underwent magnetic resonance imaging using a 3.0 Tesla scanner (MAGNETOM Trio, Siemens). The severity of stenosis in each relevant intracranial artery was graded as normal, mild stenosis (<50%), and moderate or severe stenosis (≥50%) through magnetic resonance angiography, CT angiography, or digital subtraction angiography. All radiological assessments were performed by two trained neurologists who were blind to patients’ information.

### 2.3 Statistical analysis

All statistical analyses were performed using SPSS version 26.0 (SPSS Inc.). Continuous variables with a normal distribution were presented as mean (±SD), variables with non‐normal distribution were expressed as median (interquartile range [IQR]), and categorical variables were presented as frequency (percentages). Baseline characteristics between the PI group and non‐PI group were evaluated using Student's *t*‐test, Mann–Whitney *U* test, and chi‐square test, as appropriate. In addition, the TG/HDL‐c ratio was compared among three groups according to relevant artery stenosis by the Kruskal–Wallis *H* test. Based on the results of the univariate analyses, variables with *p* < .05 were entered into the multivariable logistic regression analysis. Based on the TG/HDL‐c ratio values, we grouped all patients according to the IQR principle, and the lowest quartile was used as the reference (Q1, <1.8975; Q2, 1.8975–3.05; Q3, 3.05–4.5525; Q4, >4.5525). We used receiver operating characteristic (ROC) curves to evaluate the ability of the TG/HDL‐c ratio to predict PI in ACSSI patients and to determine the specificity, sensitivity, and optimal cutoff point. All variables with a *p*‐value <.05 were considered statistically significant.

## 3 RESULTS

**FIGURE 1:**
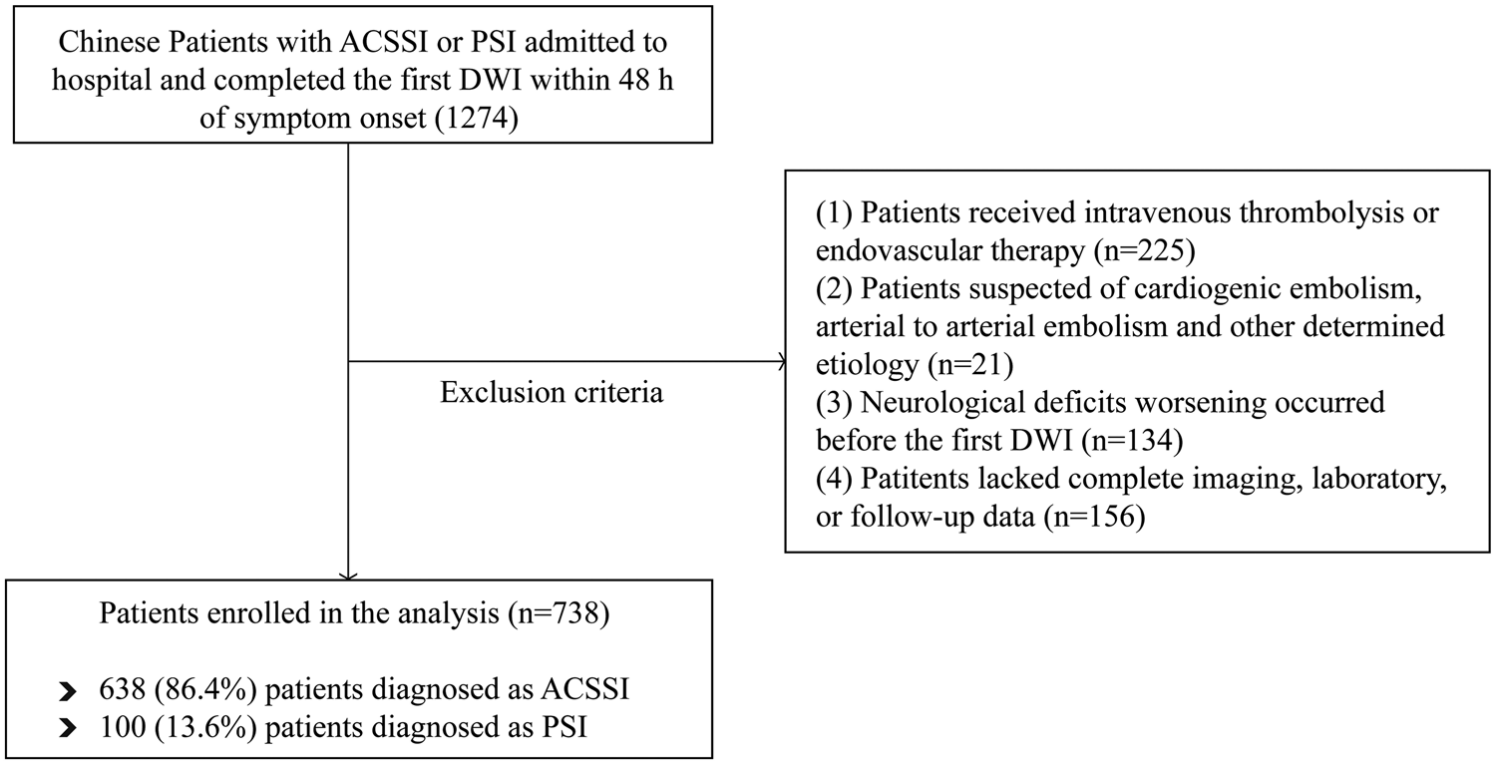
Selection of study participants.

From January 2020 and October 2022, 1274 patients who met the inclusion criteria were screened. Of those, 536 patients were excluded, 225 because of receiving intravenous thrombolysis or endovascular therapy, 21 because of cardiogenic embolism, arterial to arterial embolism, and other determined etiology, 134 because of occurring neurological deficits worsening before the first DWI, 156 because of missing complete imaging, laboratory, or follow‐up data (Figure 1). Thus, 738 patients (638 ACSSI patients and 100 PSI patients) enrolled in the analysis, including 143 patients with PI and 595 patients with non‐PI.

The baseline data between the PI and non‐PI groups in 738 patients are listed in Table 1. There were no significant differences in age, sex, history of hypertension, diabetes, stroke, infarction location, grade of relevant artery stenosis, WBC, RBC, HGB, PLB, NLR, LMR, PLR, BUN, uric acid, HDL‐c, fibrinogen, and D‐dimer (*p* > .05). However, the initial NIHSS score, discharge NIHSS score, the level of fasting glucose, the level of TC, the level of TG, the level of LDL‐c, and the level of TG/HDL‐c ratio in the PI group were higher than those in the non‐PI group (*p* < .05). In contrast, the level of creatinine in the PI group was lower compared to the non‐PI group (*p* < .05).

**TABLE 1:** Comparison of baseline characteristics between progressive infarction (PI) and non‐PI groups.

Variable	PI (*n* = 143)	Non‐PI (*n* = 595)	*P*
**Demographic characteristics**			
Age (y), median (IQR)	65 (55, 71)	64 (56, 72)	.854
Male, *n* (%)	88 (61.5%)	388 (65.2%)	.41
**Clinical and imaging data**			
Hypertension, *n* (%)	131 (91.6%)	534 (89.7%)	.503
Diabetes, *n* (%)	49 (34.3%)	201 (33.8%)	.913
History of stroke, *n* (%)			.967
None	123 (86.0%)	514 (86.4%)	
Ischemic	18 (12.6%)	71 (11.9%)	
Hemorrhagic	2 (1.4%)	10 (1.7%)	
Initial NIHSS score, median (IQR)	4 (2, 6)	3 (2, 5)	.005^*^
Discharge NIHSS score, median (IQR)	4 (2, 6)	2 (1, 3)	<.001^*^
Infarction location, *n* (%)			.497
ACSSI	121 (84.6%)	517 (86.9%)	
PSI	22 (15.4%)	78 (13.1%)	
Relevant artery stenosis, *n* (%)			.102
0	100 (69.9%)	466 (78.3%)	
<50%	30 (21.0%)	91 (15.3%)	
≥50%	13 (9.1%)	38 (6.4%)	
**Laboratory data**			
WBC (10*9/L), median (IQR)	6.70 (5.32, 7.92)	6.45 (5.23, 7.60)	.115
RBC (g/L), median (IQR)	4.43 (4.16, 4.82)	4.45 (4.15, 4.79)	.95
HGB (g/L), median (IQR)	135 (126, 145)	135 (125, 144)	.54
PLT (10*9/L), mean ± SD	204.27 ± 56.96	204.51 ± 55.98	.963
NLR, median (IQR)	2.60 (1.95, 3.75)	2.57 (1.88, 3.69)	.559
LMR, median (IQR)	3.76 (3.12, 4.75)	3.56 (2.82, 4.59)	.129
PLR, median (IQR)	126.45 (96.2, 159.41)	128.79 (97.94, 171.79)	.466
BUN (mmol/L), median (IQR)	4.5 (3.7, 5.6)	4.78 (3.80, 5.64)	.232
Creatinine (umol/L), median (IQR)	70.6 (56.3, 83.0)	74.1 (60.6, 87.6)	.048^*^
Uric acid (mmol/L), median (IQR)	337.9 (282.0, 418.0)	334.9 (278.81, 398.7)	.238
Fasting glucose (mg/dL), median (IQR)	108.84 (91.9, 141.28)	99.29 (86.14, 130.46)	.017^*^
TC (mg/dL), median (IQR)	192.58 (171.31, 225.83)	184.07 (155.84, 206.5)	.001^*^
TG (mg/dL), median (IQR)	150.62 (105.43, 194.92)	124.04 (89.49, 176.31)	.002^*^
HDL‐c (mg/dL), median (IQR)	43.7 (35.96, 50.66)	44.08 (36.35, 51.43)	.748
LDL‐c (mg/dL), median (IQR)	115.62 (99, 144.63)	111.37 (88.94, 131.48)	.002^*^
Fibrinogen (g/L), median (IQR)	2.9 (2.56, 3.4)	2.89 (2.53, 3.3)	.473
D‐dimer (mg/L), median (IQR)	0.25 (0.14, 0.48)	0.28 (0.15, 0.48)	.285
TG/HDL‐c ratio, median (IQR)	3.36 (2.12, 4.92)	2.99 (1.87, 4.32)	.011^*^

Abbreviations: ACSSI, anterior circulation single subcortical infarction; BUN, blood urea nitrogen; HDL‐c, high‐density lipoprotein cholesterol; HGB, hemoglobin; IQR, interquartile range; LDL‐c, low‐density lipoprotein cholesterol; LMR, lymphocyte‐to‐monocyte ratio; NIHSS, National Institute of Health Stroke Scale; NLR, neutrophil‐to‐lymphocyte ratio; PLR, platelet‐to‐lymphocyte ratio; PLT, platelet; PSI, pontine single infarction; RBC, red blood cell; SD, standard deviation; TC, total cholesterol; TG, triglyceride; TG/HDL‐c ratio, triglyceride to high‐density lipoprotein cholesterol ratio; WBC, white blood cell.

* *p* < .05.

As presented in Table 2, the univariate analysis revealed significant associations between PI and TC or LDL‐c, whereas there was no significant association after adjusting all variables with *p* < .05. Alternatively, the patients were divided into quartiles based on the TG/HDL‐c ratio (the first quartile was the reference value), and multivariate logistic regression analysis demonstrated that the highest TG/HDL‐c ratio quartile was independently associated with PI after adjusting age, sex, initial NIHSS score, creatinine, fasting glucose, TC, and LDL‐c (OR 1.776, 95% CI 1.022–3.085, *p* = .042) (Table 3).

**TABLE 2:** Univariate and multivariate logistic regression analysis of risk factors for progressive infarction.

Covariate	Univariate		Multivariate	
	OR (95% CI)	*p* Value	OR (95% CI)	*p* Value
Initial NIHSS score	1.056 (.997–1.119)	.063	1.047 (.987–1.110)	.13
Creatinine	.993 (.985–1.001)	.098	.993 (.985–1.001)	.1
Fasting glucose	1.002 (1.000–1.005)	.104	1.001 (.998–1.004)	.606
TC	1.006 (1.002–1.01)	.005^*^	.997 (.987–1.008)	.64
LDL‐c	1.008 (1.002–1.013)	.004^*^	1.010 (.997–1.022)	.128
TG/HDL‐c ratio	1.042 (.988–1.099)	.13	1.055 (.991–1.124)	.094

Abbreviations: CI, confidence interval; LDL‐c, low‐density lipoprotein cholesterol; NIHSS, National Institute of Health Stroke Scale; OR, odds ratio; TC, total cholesterol; TG/HDL‐c ratio, triglyceride to high‐density lipoprotein cholesterol ratio.

* *p* < .05.

**TABLE 3:** Evaluation of the impact of triglyceride to high‐density lipoprotein cholesterol (TG/HDL‐c) ratio on progressive infarction after grouping patients by TG/HDL‐c ratio quartiles.

	OR	95% CI	*p*
Adjusted model^a^			
TG/HDL‐c ratio Q1 (reference)			
Q2	1.276	.734–2.219	.388
Q3	1.502	.874–2.581	.141
Q4	1.837	1.073–3.146	.027^*^
Adjusted model^b^			
TG/HDL‐c ratio Q1 (reference)			
Q2	1.255	.718–2.194	.426
Q3	1.399	.809–2.420	.230
Q4	1.74	1.013–2.989	.045^*^
Adjusted model^c^			
TG/HDL‐c ratio Q1 (reference)			
Q2	1.265	.722–2.217	.410
Q3	1.417	.816–2.459	216
Q4	1.776	1.022–3.085	.042^*^

Abbreviations: CI, confidence interval; LDL‐c, low‐density lipoprotein cholesterol; NIHSS, National Institute of Health Stroke Scale; OR, odds ratio.

^a^ Adjusted model was controlled for age, sex.

^b^ Adjusted model was controlled for initial NIHSS score, creatinine, fasting glucose, total cholesterol, LDL‐c.

^c^ Adjusted model was controlled for age, sex, initial NIHSS score, creatinine, fasting glucose, total cholesterol, LDL‐c.

* *p* < .05.

The results of the subgroup analyses are shown in Table 4. The TG/HDL‐c ratio was an independent risk factor for PI in patients with ACSSI (OR 1.079, 95% CI 1.009–1.153, *p* = .026), but there was no correlation in patients with PSI (*p* > .05). The ROC curve was further to estimate the predictive value of the TG/HDL‐c ratio on PI. For ACSSI patients, we observed that the area under the curve was 0.592 (95% CI .537–.647), and the best predictive cutoff value was 3.985, with a sensitivity of 41.3% and a specificity of 74.1%. When we divided ACSSI patients into two groups around the cutoff value, the frequency of PI was 15.6% in patients with a TG/HDL‐c ratio <3.985 and 27.2% in patients with a TG/HDL‐c ratio ≥3.985 (*p* = .001). Furthermore, there was a significant statistical difference among the three groups according to relevant artery stenosis in ACSSI patients [0 stenosis, median (IQR), 2.90 (1.78, 4.34); <50% stenosis, median (IQR), 2.99 (1.87, 4.21); ≥50% stenosis, median (IQR), 3.93 (2.78, 5.52); *p* = .005].

**TABLE 4:** Multivariate logistic regression analysis of risk factors for progressive infarction in anterior circulation single subcortical infarction (ACSSI) subgroup and pontine single infarction (PSI) subgroup.

Covariate	ACSSI (*n* = 638)		PSI (*n* = 100)	
	Adjusted OR (95% CI)	*p* Value	Adjusted OR (95% CI)	*p* Value
Initial NIHSS score	1.067 (1.003–1.136)	.039^*^	.882 (.721–1.079)	.222
TC	.995 (.984–1.007)	.413	1.018 (.985–1.052)	.29
LDL‐c	1.013 (.999–1.026)	.062	.991 (.953–1.031)	.656
TG/HDL‐c ratio	1.079 (1.009–1.153)	.026^*^	.848 (.688–1.046)	.123

Abbreviations: CI, confidence interval; LDL‐c, low‐density lipoprotein cholesterol; NIHSS, National Institute of Health Stroke Scale; OR, odds ratio; TC, total cholesterol; TG/HDL‐c ratio, triglyceride to high‐density lipoprotein cholesterol ratio.

* *p* < .05.

## 4 DISCUSSION

The present study showed that elevated TG/HDL‐c ratio was an independent predictor for PI in patients with acute ischemic stroke within 48 h after onset, especially in patients with ACSSI. Of note, the cutoff value of the TG/HDL‐c ratio to predict PI was 3.985, and the TG/HDL‐c ratio ≥3.985 increased the risk of PI in patients with ACSSI.

Many previous studies have demonstrated that various contributors participated in the END such as initial NIHSS score, blood pressure variability, blood glucose, and so on (Kang et al., 2017; Kim et al., 2016; Yu et al., 2022). However, the current factors to predict anterior circulation SSI and posterior circulation SSI are different, partially contributing to the anatomic positional relationship (Nam et al., 2021). Therefore, we recruited patients diagnosed as ACSSI or PSI (the most common type of SSI in posterior circulation) and analyzed the relationship between TG/HDL‐c ratio and PI in these two subgroups. More importantly, a variety of mechanisms were involved in END, and we selected PI as the outcome variable rather than END in our study, reducing the impact of other END mechanisms on the conclusions.

The gold standard measurement of IR by hyperinsulinemic‐glycemic clamp technique is not suitable for daily clinical work because of its complexity and high cost (De Koster et al., 2016; Volpe & Sotis, 2015). Instead, the homeostasis model assessment for IR (HOMA‐IR) index is widely used to evaluate IR in children, adolescents, and adults (Geloneze et al., 2009; Tresaco et al., 2005). Nevertheless, the TG/HDL‐c ratio, as a novel predictor or marker of IR, has been validated as a better indicator than the HOMA‐IR to screen for a positive diagnosis of metabolic syndrome (Liang et al., 2015). Given the routine and inexpensive tests of TG and HDL‐c, the TG/HDL‐c ratio is a useful tool for assessing IR in clinical practice (Collins et al., 2017). There have been increasing studies demonstrating the associations of TG/HDL‐c ratio with metabolic diseases and cardiovascular disease (Che et al., 2023; Chen et al., 2022; Jialal et al., 2022). In addition, recent evidence indicates that TG/HDL‐c ratio is associated with a higher risk of stroke and the recurrence of stroke (Nam et al., 2019; Park et al., 2014). To our knowledge, this is the first report to explore the correlation between TG/HDL‐c ratio and PI and demonstrates that increased TG/HDL‐c ratio was an independent predictor of PI.

The mechanism underlying the relationship between IR and PI is unclear. Many previous studies have indicated that IR plays an important role in the development of atherosclerosis and advanced plaque, by promoting elevated synthesis and release of lipoproteins, and the growth of vascular smooth muscle cells (Di Pino & DeFronzo, 2019; Pané et al., 2020; Yamazoe et al., 2016). In the past, PI had been proposed to be attributed to clot progression (Thanvi et al., 2008). More importantly, our findings revealed that the TG/HDL‐c ratio gradually increased with the severity of relevant artery stenosis, indicating the relationship between the TG/HDL‐c ratio and intracranial atherosclerotic stenosis. Therefore, we speculate that the correlation between elevated TG/HDL‐c ratio and PI is through plaque progression.

Although our study contains interesting novel findings, there are some limitations in our study. First, this is a single‐center retrospective study of patients with a single ethnic background. However, the detection of laboratory parameters using the same method in a stroke center results in better uniformity. Second, the sample size of PSI is a little bit small. Future studies should further expand the sample size of PSI and collect multicenter and multiethnic patients to confirm the findings.

## 5 CONCLUSION

In conclusion, our study demonstrated TG/HDL‐c ratio as a predictor of PI in patients with ACSSI.

## AUTHOR CONTRIBUTIONS

**Jing Lin**: Conceptualization; writing—original draft; funding acquisition. **Liangbin Dong**: Conceptualization; writing—original draft. **Qin Huang**: Methodology; data curation; formal analysis. **Hui Xiao**: Methodology; data curation; formal analysis. **Shumeng Li**: Methodology; data curation; formal analysis. **Jincai Tang**: Methodology; data curation; formal analysis. **Xiaocheng Mao**: Methodology; data curation; formal analysis. **Pengcheng Huang**: Methodology; data curation; formal analysis. **Xiaobing Li**: Conceptualization; writing—review and editing; supervision. **Daojun Hong**: Conceptualization; funding acquisition; supervision; writing—review and editing.

## CONFLICT OF INTEREST STATEMENT

The authors declare that there is no conflicts of interest.

### PEER REVIEW

The peer review history for this article is available at https://publons.com/publon/10.1002/brb3.3453.

## Data Availability

Data are available upon reasonable request. All data are available from the corresponding author.
